# “Hospital survival of patients with pulmonary embolism in a country with limited resources case of the city of Kinshasa”

**DOI:** 10.1186/s12872-023-03467-6

**Published:** 2023-09-04

**Authors:** Tshilanda Balekelayi Marc, Tshiasuma Pipo Michel, Mpembe Florence, Mujijo Tousaint, Kazadi Serge, Bosenedje Chadrack, Kokusa Roly, Dizolele Gédéon, Makanzu Madioko Brady, Kamangu Ntambue Erick

**Affiliations:** 1 Department of Internal Medicine, Faculty of Medicine, Notre-Dame du Kasayi University, Kananga, Congo; 2 Department of Internal Medicine, Center Hospitalier Mère-Enfant Monkole, Kinshasa, Congo; 3 Department of Internal Medicine, Faculty of Medicine, University Clinics of Kinshasa, University of Kinshasa, Kinshasa, Congo; 4 Department of Internal Medicine, Faculty of Medicine, Simon-Kimbangu University, Kinshasa, Congo; 5 Department of Basic Sciences, Faculty of Medicine, University of Kinshasa, Kinshasa, Congo

**Keywords:** Survival, In-hospital, Pulmonary embolism, Resource-limited country, Kinshasa

## Abstract

**Background:**

Pulmonary embolism is a frequent cause of intra-hospital mortality. The survival of patients depends not only on the speed of diagnosis but also on the treatment initiated.

**Objective:**

to evaluate the intra-hospital survival of patients with pulmonary embolism in the city of Kinshasa.

**Methodology:**

analytical cross-sectional study based on non-probability convenience sampling carried out in the city of Kinshasa; three hospitals selected for convenience on the basis of their technical platforms. The patients were judged to have had a pulmonary embolism after a chest CT angiography proved it. The data recorded on the Kobocollect site were exported in Excel format and analyzed with SPSS software version 23. The comparison of the means was made using the Student test and that frequencies with the Yates Chi-square test. The association was attributed by the calculation of the odds ratio and the survival presented according to the Cox regression.

**Results:**

Eighty-nine cases or 63 women and 26 men were analyzed, the mean age was 64.4 ± 15.6 years. Individuals over 65 died more (ß=0.043 and p-Value of 0.01) the female sex multiplied by 1.38 the risk of death (DNS, p-Value = 0.478). Approximately 80% of patients were classified as PESI stage II or III. Starting Rivaroxaban from the outset does not show any difference with enoxaparin in terms of intra-hospital survival.

**Conclusion:**

Pulmonary embolism is a real problem in our environment, the age of more than 65 years and the female sex are factors of poor prognosis and predicted survival.

## Introduction

Pulmonary embolism is defined as the sudden, partial or total obstruction of a pulmonary artery or its branches by a clot. It is a frequent pathology whose incidence increases with age. In older subjects over the age of 75, its incidence reaches one case per 100 people per year [1].

The pathology being of varied clinical presentation despite the high mortality rate, requires rapid diagnosis and early treatment. Treatment is also cumbersome for severe forms of the disease; he therefore resorts to anticoagulants and may require emergency thrombolysis. Several treatment regimens have been proposed for the management of pulmonary embolism; one who uses low molecular weight heparin relay with an Anti-vitamin K and the other who uses oral anticoagulants from the outset for three to six weeks [1, 2].

Pulmonary embolism is considered the third cardiovascular disease in the United States, with a crude mortality rate in France of 22.9 per 100,000 from multiple causes and an incidence of 60/100,000 for pulmonary embolism [2]. In Africa, general data on this subject are difficult to find in the literature, nevertheless certain hospital studies attest to the existence of the pathology with a very high mortality. At the CHU du point G in Bamako, Mali, Coulibaly reports a hospital frequency of VTE of 4.95% with 1.8% for pulmonary embolism [3]. In the DRC (Democratic Republic of Congo), a study conducted in 2015 by Mboliasa et al. on the epidemiological and clinical profile of cardiovascular emergencies admitted to the intensive care unit of the Internal Medicine of the university clinics of Kinshasa had noted a hospital frequency of pulmonary embolism of 11.3% [4].

The different treatment regimens for pulmonary embolism are rarely compared according to the characteristics of certain populations in terms of their benefits on survival. However, some practitioners in developing countries are increasingly using low molecular weight heparins.

This work has set itself the objective of evaluating the intra-hospital survival of patients with pulmonary embolism according to the care in the city of Kinshasa.

## Methodology

Our study was conducted in three secondary hospitals in the city of Kinshasa, capital of the Democratic Republic of Congo. This city consists of 35 health zones for a coverage of approximately 17 million inhabitants. The hospitals concerned were respectively Ngaliema Clinic (C. Ngaliema), Biamba Marie Mutombo Hospital (HBMM) and Monkole Mother-Child Hospital Center (CH. Monkole) selected by convenience on the basis of their technical platforms.

We conducted an analytical cross-sectional study based on non-probability convenience sampling for the selection of 89 patients who met our selection criteria. Adult patients (over 18 years old) who consulted in the selected hospital from January 1, 2018 to December 31, 2021 and whose chest CT angiography had revealed a pulmonary embolism were included in the study. Any patient suspected of pulmonary embolism who had not performed a chest CT angiography was excluded.

The parameters studied were essentially socio-demographic data (age, sex and marital status), history, clinical signs, anthropometric data and vital signs, clinical probability, treatments administered, clinical evolution and the hospital stay.

The collection of data was carried out from sheets, registers of patients as well as computer software for the management of the person treated for the case of CH Monkole. The data was encoded with the smartphone on the Kobbocollect application from a pre-established questionnaire. The data recorded on the Kobocollect site were exported in Excel format and analyzed with SPSS version 23 software. The simplified Sore Pulmonary Embolism Severity Index (sPESI) was used to determine the severity of the disease in terms of risk of recurrence, patients with a score less than 1 were classified with low risk of recurrence and those with a score greater than or equal to 1 with high risk.

The data normalized with a Skweness test at 0.85 with a confidence interval of -1.2 to 1.65 and a Kurtosis test at 1.85 were represented in the form of tables, translated into frequency and relative numbers.

The comparison of the means was made using the Student test and that of the frequencies with the Yates Chi-square test.

The analysis consisted in the calculation of central frequencies and tendencies, the calculation of the risk to establish the association of the treatment and post-therapeutic evolution. Survival was presented on the Cox regression model.

## Results

**Table 1:** General patient characteristics

	Total n=89	CH Monkole n=24	HBMM n=36	C. Ngaliema n=29
**Age (in years)**	64.4 ± 15.6	60.1 ± 15.7	63.3 ± 15.3	69.2 ± 15.1
**Gender**				
Feminine	63 (70.8%)	18 (75%)	23 (63.9%)	22 (75.9%)
Male	26 (29.2%)	6 (25%)	13 (36.1%)	7 (24.1%)
**Civil state**				
Bachelor	2 (2.2%)	1 (4.2%)	0 (0.0%)	1 (3.4%)
Divorced	3 (3.4%)	0 (0.0%)	0 (0.0%)	3 (10.3%)
Married	50 (56.2%)	15 (62.5%)	24 (66.7%)	11 (37.9%)
widow/widower	34 (38.2%)	8 (33.3%)	12 (33.3%)	14 (48.3%)
**Antecedents**				
High blood pressure	61 (68.5%)	15 (62.5%)	27 (75%)	19 (65.5%)
Diabetes	25 (28.1%)	2 (8.3%)	15 (41.7%)	8 (27.6%)
Progressive cancer	4 (4.5%)	2 (8.3%)	0 (0.0%)	2 (6.9%)
Deep vein thrombosis (DTV)	6 (6.7%)	4 (16.7%)	1 (2.8%)	1 (3.4%)
Pulmonary embolism (PE)	18 (20.2%)	8 (33.3%)	0 (0.0%)	10 (34.5%)
Surgery less than a week	17 (19.1%)	3 (12.5%)	6 (16.7%)	8 (27.6%)
Stroke	5 (5.6%)	1 (4.2%)	0 (0.0%)	4 (13.8%)
**Symptoms**				
Fever	15 (16.9%)	4 (16.7%)	8 (22.2%)	3 (10.3%)
Chest pain	27 (30.3%)	4 (16.7%)	6 (16.7%)	17 (58.6%)
Dyspnea	69 (77.5%)	20 (83.3%)	25 (69.4%)	24 (82.8%)
Physical asthenia	22 (24.7%)	4 (16.7%)	7 (19.4%)	11 (37.9%)
Lower limb pain	10 (11.2%)	2 (8.3%)	7 (19.4%)	1 (3.4%)
Swelling of the lower limbs	9 (10.1%)	3 (12.5%)	5 (13.9%)	1 (3.4%)
**Physical examination**				
Systolic blood pressure in mmHg	133.7 ± 31.01	137.7 ± 36.4	129.1 ± 28.6	139.9 ± 27.7
Diastolic blood pressure in mmHg	78.7 ± 17.5	82.9 ± 23.5	71.9 ± 13.2	83.6 ± 14.06
Body mass index in Kgs/m2	25.4 ± 4.5	27.7 ± 6.3	25.3 ± 3.9	23.8 ± 2.6
Cardiac frequency /min	92 ± 16.9	95.7 ± 19.08	92.06 ± 17.5	91.3 ±14.3
Respiratory rate /min	25.2 ± 6.08	28.3 ± 8.4	25.1 ± 4.1	22.7 ± 4.6
Peripheral oxygen saturation in %	89.8 ± 12.3	84.1 ± 15.8	92.5 ± 12.49	91.07 ± 5.8
Temperature in °C	36.4 ± 0.64	36.3 ± 0.6	36.36 ± 0.68	36.6 ± 0.62
**Clinical probability (modified and simplified Geneva score)**				
Weak	31 (16.3%)	2 (8.3%)	5 (13.9%)	7 (24.1%)
Strong	53 (27.9%)	4 (16.7%)	13 (36.1%)	11 (37.9%)
Intermediate	106 (55.8%)	18 (75%)	18 (50%)	11 (37.9%)
**D-dimer**	5171.4 ± 478.7	2740.4 ± 669.8	2443.2 ± 455.2	9680.5±1088.3
**Venous Doppler ultrasound of the lower limbs**				
DVT-	36(40.4%)	11(45.8%)	9(25.0%)	16(55.1%)
DVT+	53(59.6%)	13(54.2%)	27(75.0%)	13(44.9%)

The average age is 64.4 ± 15.6 overall with a female predominance with a sex ratio of 0.7 (Table 1). Six out of 10 patients are married overall. Two out of ten patients have a history of pulmonary embolism and 6% a history of DVT. Dyspnea is the most common symptom, found in 77.5% of cases. The mean arterial pressure remained within the norms 133.7 ± 31.01 out of 78.7 ± 17.5 with a normal heart rate of 92.8 ± 16.9 bpm and a collapsed peripheral oxygen saturation at 89.8 ± 12. 0.3%. Six out of ten patients had an intermediate clinical probability with D-dimers at 5171.4 ± 478.7 ng/L. Six out of ten patients had DVT on Doppler ultrasound of the lower limbs.

**Table 2:** Treatment administered

	Total n=89	CH Monkole n=24	HBMM n=36	C. Ngaliema n=29
Hospital stay (day)	8.0 ± 1.12	7.3 ± 1.09	7.25 ± 0.71	9.4 ± 0.85
Hospital stay interval (day)	[1-26]	[0-26]	[2–18]	[2–15]
Pulmonary Embolism Severity Index				
high	9(10.1%)	2(8.3%)	5(13.9%)	2(6.9%)
Low-intermediate	41(46.1%)	5(20.8%)	25(69.4%)	11(37.9%)
Upper middle	39(43.8%)	17(70.8%)	6(16.7%)	16(55.2%)
Treatment administered				
Thrombolysis	2 (2.2%)	0(0.0%)	0(0.0%	2(6.9%)
Enoxaparin	75 (84.3%)	10(41.7%)	36(100%)	29(100.0%)
Rivaroxabam	48(53.9%)	16(66.7%)	26(72.2%)	22(75.9%)
Acenocoumarol	44(46.1%)	8(33.3)	10(27.8%)	7(24.1)
Remedy treatment within the first 7 days				
Rivaroxaban from the outset	14(15.7%)	9(37.5%)	3(8.3%)	2(6.6%)
Enoxaparin from the start	75(84.3%)	15(62.5%)	33(91.7)	27(93.1%)
Hospital outcome				
Death	29 (32,6)	7(29.2%)	11(30.6%)	11(37.9%)
Healing	60 (67.4%)	17(70.8)	25(69.4%)	18(62.1%)
Deaths over time (n=29)				
<8 days	23 (79.3%)	7 (100.0%)	8 (72.7%)	8 (72.7%)
8-20 days	6 (20.7%)	0 (0.0%)	3 (27.3%)	3 (27.3%)
>20 days	0 (0.0%)	0 (0.0%)	0 (0.0%)	0 (0.0%)
Death by sex				
Male	22 (75.9%)	5 (71.4%)	10 (90.9%)	7(63.6%)
Feminine	7 (24.1%)	2(28.6%)	1 (9.1%)	4 (36.4%)
Death by age				
<65 years	4 (13.8%)	1 (14.3%)	2 (18.2%)	1 (9.1%)
≥65 years	25 (96.2%)	6 (85.3%)	9 (81.2%)	10(90.9%)

Patients experienced an average of 8.0 ± 1.12 hospital days with a range of 0 to 26 hospital days; Half of the patients had a low intermediate risk against 10% of the high risk, of which only 2 cases, or 2% of the general population, received thrombolysis as the first treatment administered (Table 2). Eight out of ten patients received at least enoxaparin in their treatment. Fourteen patients, or 15.7%, immediately started treatment with Rivaroxaban. Intra-hospital mortality was estimated at 32.6% of cases including 79.3% before 8 days of hospitalization, 20.7% between 8 and 20 days. No deaths were recorded in patients who had been hospitalized for more than 20 days. Twenty-two patients or 75.9% were male with 96% of patients over 65%.

**Table 3:** Factor associated with death

	ß	OR (IC95%)	p-Value
Age over 65	0.043	1.044 (1.01-1.079)	0.01
Female gender	0.322	1.38 (0.56-3.36)	0.478
POS less than 85%	-0.027	0.973 (0.94-1)	0.082
h greater than 94bm	0.013	1.013 (0.99-1.03)	0.267
High D-dimer	0	1	0.792
Type of treatment initiated immediately	-0.378	0.685 (0.20-2.27)	0.537

Age over 65 is associated with death (ß=0.043 and p-Value of 0.01) female sex multiplies the risk of death by 1.38 (DNS, p-Value = 0.478), the other factors are weakly associated with risk of intra-hospital mortality (Table 3).

**Fig. 1:**
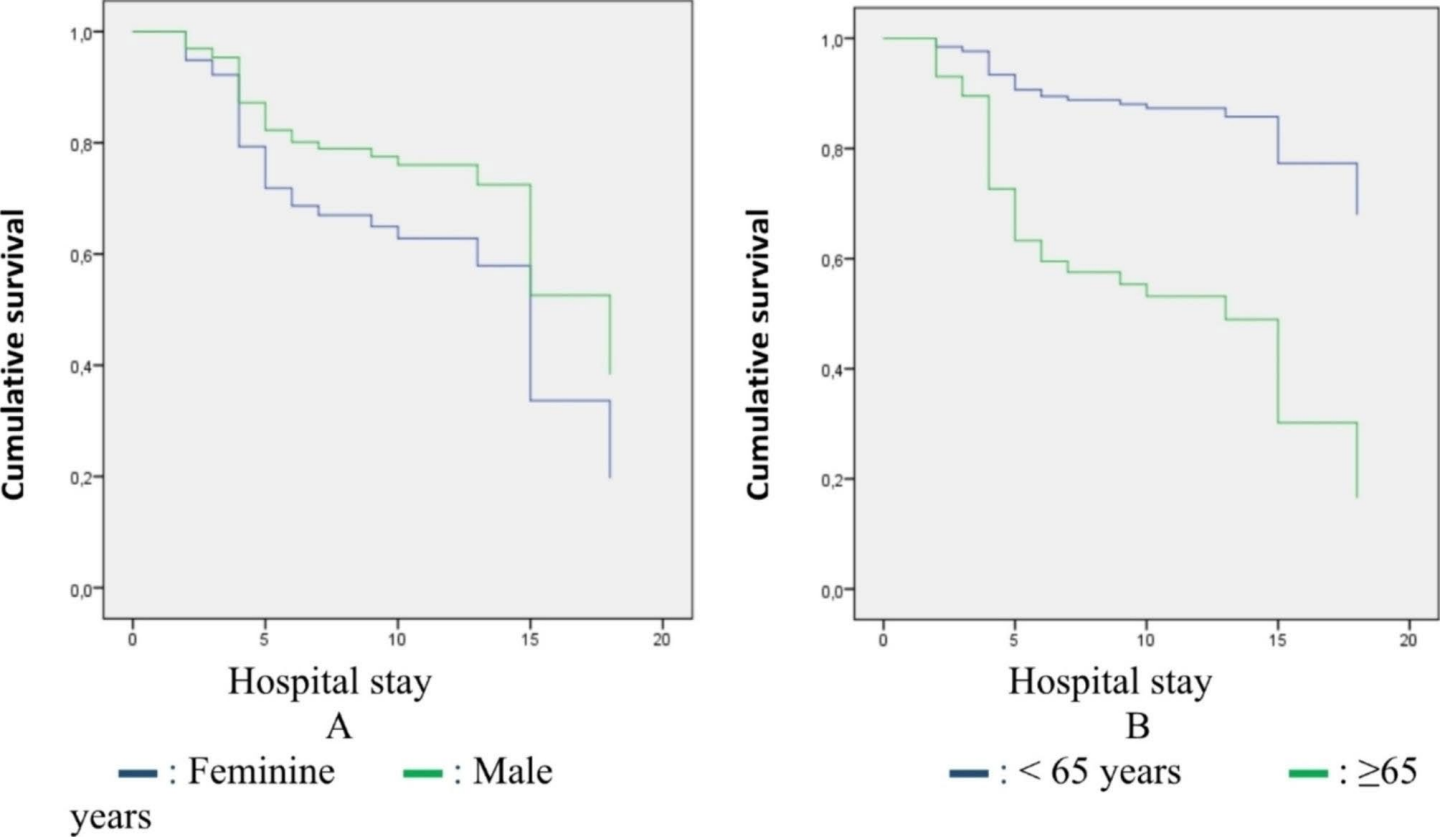
Patient survival by gender (**A**) Survival by age (**B**). Patients under 65 years old have a good survival compared to patients over 65 years old with the same evolution in both sexes with regard to survival

**Fig. 2:**
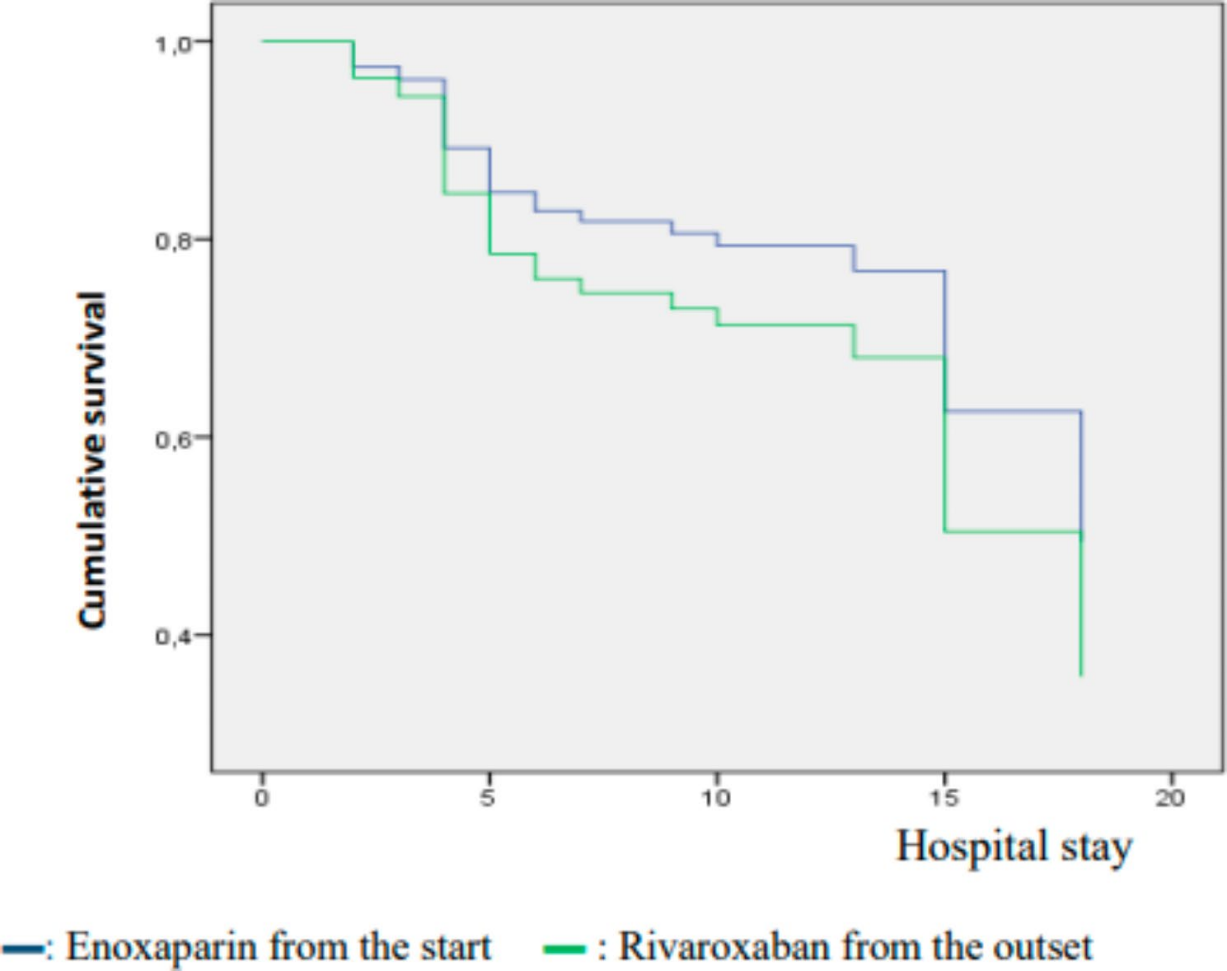
Survival by type of treatment administered. Starting on Rivaroxaban from the outset presents a similar evolution to starting on Enoxaparin in terms of intra-hospital survival of patients

## Discussion

Our sample consisted of 63 women and 26 men who had an average age of 64.4 ± 15.6 years overall. In the series by Pessinaba S et al. the mean age of the patients was 52.7 ± 14.4 years with a hospital prevalence of 3.1% and a female predominance (F/M = 2.2) [5]. Konin noted an average age of 48.5 years at the Abidjan Cardiology Institute [6]. Two out of ten patients have a history of pulmonary embolism and 6% a history of DVT. Dyspnea is the most common symptom, found in approximately 8 out of 10 patients. Six out of ten patients had DVT on Doppler ultrasound of the lower limbs. In the series by Pessinaba S et al. VTE risk factors were dominated by obesity (54.9%), bed rest (25.5%) and long travel (17.6%) [5].

The hospital stay was on average 8.0 ± 1.12 days of hospitalization; Half of the patients had a low intermediate risk (Stage II) against 10% high risk (Stage III-IV) according to the prognostic Sore of the Pulmonary Embolism Severity Index, results close to those of the Bakebe series [7] i.e. 7% of the cases classified III-V. Hospital stay was short in Konin’s series at 5.2 days [6].

Intra-hospital mortality was estimated at 32.6% of cases. Eight out of 10 patients died before 8 days of hospitalization and two out of 10 patients died between 8 and 20 days, 96% of whom were over 65 years old. No deaths were recorded in patients who had been hospitalized for more than 20 days. In the series by Pessinaba S et al. the treatment consisted of low molecular weight heparin at a curative dose followed by an AVK. Thrombolysis was performed in 2 patients. The outcome was favorable in 86.3%. The case fatality rate was 13.7% [5]. The Pessinaba series involved 51 patients treated in a single center with a single treatment protocol, the mortality of this series is about half of ours, a difference probably linked to the different characteristics of the two samples and different research methods [7].

Age over 65 is associated with death (ß=0.043 and p = 0.01), being female increases the risk of death by 1.38 (DNS, p = 0.478), other factors are weakly associated with the risk of death intra-hospital. In the Konin series, the excess mortality factors were essentially cancer (P = 0.02), right heart failure (P = 0.04), cardiogenic shock (P < 0.0001), desaturation (SatO2 < 90%) (P < 0.0001), sinus tachycardia (P = 0.02). Desaturation below 85% and sinus tachycardia over 94 beats per minute did not appear in our series sum of risk factors (OR: 0.97 and 1.013; p: 0.082 and 0.267) [6]. It is important to note that in our series the average oxygen saturation was around 90% and the heart rate at 92 bpm.

Patients under 65 have good survival compared to patients over 65. Gender does not influence the survival of patients with embolism in our series as shown in Fig. 1 of our results. Figure 2 shows that only two patients or 2.2% of cases were treated with thrombolysis, 84.3% with enoxaparin, 53.9% rivaroxabam and 46.1% acenocoumarol. In the series by Bakebe A et al. all patients were treated with anticoagulants; no case received thrombolytics [7]. Starting Rivaroxaban from the outset shows a similar evolution to enoxaparin in terms of intra-hospital survival of patients, like the data from the EINSTEIN-PE study where rivaroxaban did not appear lower than the standard diet for the treatment of pulmonary embolism [8].

## Conclusion

Pulmonary embolism is a serious disease with a poor prognosis. The factors associated with mortality are multiple and make the prognosis poor. The female sex, the advanced age of more than 65 years emerged from our series as being the most associated with mortality. Survival is lower in individuals over 65 years of age.

## Data Availability

The datasets used and/or analysed during the current study are available from the corresponding author on reasonable request.
